# MiR-675-5p supports hypoxia induced epithelial to mesenchymal transition in colon cancer cells

**DOI:** 10.18632/oncotarget.14464

**Published:** 2017-01-03

**Authors:** Viviana Costa, Alessia Lo Dico, Aroldo Rizzo, Francesca Rajata, Marco Tripodi, Riccardo Alessand, Alice Conigliaro

**Affiliations:** ^1^ Innovative Technological Platforms for Tissue Engineering, Theranostic and Oncology, Rizzoli Orthopedic Institute, Palermo, Italy; ^2^ Department of Pathophysiology and Transplantation, Università degli Studi di Milano, Milano, Italy; ^3^ Unità Operativa di Anatomia Patologica, Azienda Ospedaliera Ospedali Riuniti “Villa Sofia-Cervello”, Palermo, Italy; ^4^ Dipartimento di Biotecnologie Cellulari ed Ematologia, Sapienza University of Rome, Rome, Italy; ^5^ National Institute for Infectious Diseases L. Spallanzani, IRCCS, Rome, Italy; ^6^ Dipartimento di Biopatologia e Biotecnologie Mediche, University of Palermo, Palermo, Italy; ^7^ Institute of Biomedicine and Molecular Immunology (IBIM), National Research Council of Italy, Palermo, Italy

**Keywords:** miRNA675, CRC, EMT, metastasis, hypoxia

## Abstract

The survival rates in colon cancer patients are inversely proportional to the number of lymph node metastases. The hypoxia-induced Epithelial to Mesenchymal Transition (EMT), driven by HIF1α, is known to be involved in cancer progression and metastasis. Recently, we have reported that *miR-675-5p* promotes glioma growth by stabilizing HIF1α; here, by use of the syngeneic cell lines we investigated the role of the miR-675-5p in colon cancer metastasis.

Our results show that *miR-675-5p*, over expressed in metastatic colon cancer cells, participates to tumour progression by regulating HIF1α induced EMT. *MiR-675-5p* increases Snail transcription by a dual strategy: i) stabilizing the activity of the transcription factor HIF1α and ii) and inhibiting Snail's repressor DDB2 (Damage specific DNA Binding protein 2).

Moreover, transcriptional analyses on specimens from colon cancer patients confirmed, *in vivo*, the correlation between *miR-675-5p* over-expression and metastasis, thus identifying miR-675-5p as a new marker for colon cancer progression and therefore a putative target for therapeutic strategies.

## INTRODUCTION

Colorectal Cancer (CRC) is one of the most common cancer diagnosed worldwide and still the most frequent cause of cancer-related mortality due to the development of metastases [1]. The complex set of events, driving the dissemination of single carcinoma cells from primary epithelial tumours, involves the phenotypic conversion commonly known as Epithelial to Mesenchymal Transition (EMT). [2]. Hallmark of the EMT process is considered the functional loss of E-cadherin operated by the Snail family: a family of zinc-finger transcription factors including Snail and Slug that, directly binding the E-cadherin promoter, repress gene transcription and stimulate the initial invasion [3–5]. Both Snail and Slug proteins have been implicated in various malignancies and associated with poor prognosis in carcinoma [6–9]. In colon cancer, Snail was found over expressed in human biopsies [10] while Slug expression has been proposed as a significant parameter of poor prognosis [11]. It is widely accepted that colon cancer progression and its metastasis are driven by a complex set of events and, between these, a pivotal role is played by the hypoxia in tumour microenvironment [12]. The reduced O_2_ partial pressure in tumour microenvironment, prompts tumour cell to activate specific pathways that, driven by the transcription factor Hypoxia-inducible factor 1-alpha (HIF1α), induce hypermetabolism to favour glycolysis, resistance to chemotherapy, neo-angiogenesis with an increase in local vasculature and finally, tumour metastasis. Hypoxia induces EMT in a variety of cancers including colon carcinoma [13, 14] moreover, functional Hypoxia Response Elements (HREs) have been identified within Snail's regulatory sequences [15].

Among the targets of HIF1α is the lncRNA H19 [16], an imprinted non coding RNA which expression was found up-regulated in many tumours including CRC, hepatocellular carcinoma, testicular cancer, choriocarcinoma, osteosarcoma, esophageal cancer and glioma [17–20]. As already described for other lncRNAs, H19 can work as a microRNA sponge or epigenetic modulator [21, 22] moreover, it is a reservoir for microRNA-675 (miR-675-5p and miR-675-3p) [16] by which H19 seems to promote tumour growth and metastasis [23, 24]. Recently we demonstrated, in a glioma pre-clinical model, a loop between HIF1α and miR-675-5p. We found that *miR-675-5p i)* is over expressed in hypoxic condition, *ii)* it is essential to sustain hypoxic responses for its role in HIF1α stabilization, and in particular *iii)* it promotes hypoxia-mediated angiogenesis [25].

Several miRNAs have been found aberrantly expressed in colon cancer [26] and supposed as potential markers in diagnosis, prognosis and treatment of CRC [27].

In this study, we investigated the relationship between *miR-675-5p*, hypoxia and tumour metastasis in colon cancer. Our data indicated that *miR-675-5p* maintains a metastatic phenotype through a lncRNA H19 independent mechanism. In particular, *miR-675-5p* was found over expressed in metastatic colon cancer patients while, its silencing, induced *in vitro* the inhibition of the HIF1α guided EMT, indicating the *miR-675-5p* as a new putative target and predictive marker in colon cancer.

## RESULTS

### MiR-675-5p inhibitor reduces metastatic phenotype in SW620 cells

**Figure 1:**
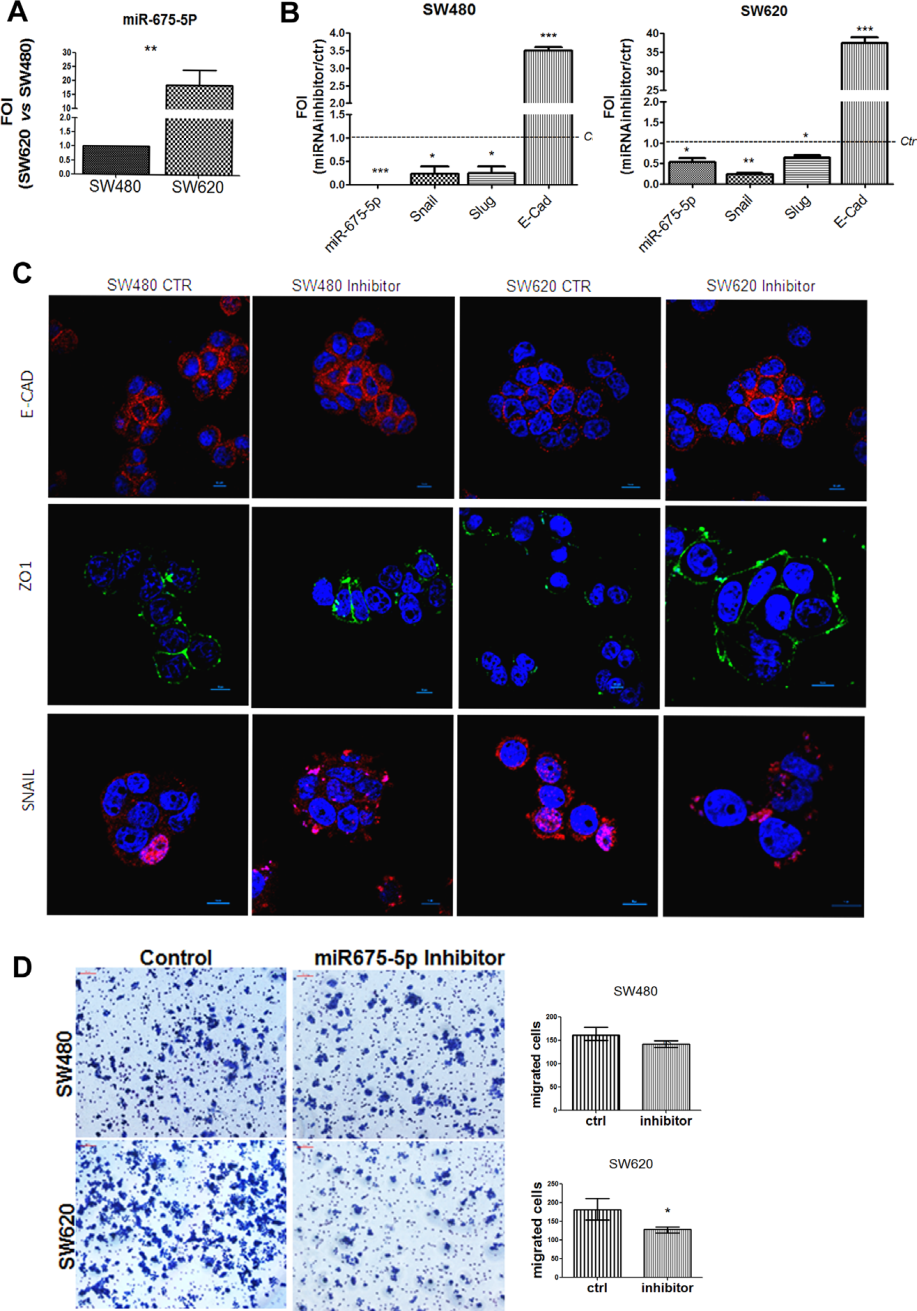
miR-675-5p inhibitor reduces metastatic features and promotes epithelial phenotype (**A**) Real time-PCR for miR-675-5p in SW480 and SW620 cells. All data were normalized for U6 and ΔΔct was expressed as relative amount of miRNAs. Values are presented as the mean ± SD (of three independent experiments). SW620 *vs* SW480 **p < 0.001. (**B**) Real time-PCR for miR-675-5p and EMT master genes: Snail, Slug and E-cadherin in SW480 and SW620 cells. Data were normalized for β-actin, while U6 was used for miRNA normalization. ΔΔct is expressed as Fold of induction (FOI) with respect to expression in control samples. Data are the mean ± SD of three independent experiments. SW480 miRNA inhibitor *vs* SW480 scramble control: *p < 0.05; ***p < 0.0001; SW620 miRNA inhibitor *vs* SW620 scramble control *p < 0.05; **p < 0.001; ***p < 0.0001. (**C**) Immunofluorescences and median focal plane in confocal analysis for E-CADHERIN, ZO-1and SNAIL in SW480 and SW620 cells treated with miR-675-5p inhibitor or scramble control, in blue the nuclear staining with DAPI. (**D**) Migration assay: Phase contrast micrographs (10×) showing the migration of SW480 and SW620 cells pre-treated with miR-675-5p inhibitor or scramble control. Right panel: Quantification of migration by counting the number of migrated cells (violet) per field (*n* = 6); *p < 0.05.

With the aim to explore a role of *miR-675-5p* in colon cancer, we took advantage of the syngeneic cell lines, SW480 and SW620, that, derived respectively from primary tumour and lymphonodal metastasis, are an *in vitro* validated model to study tumour colon progression [28]. The analysis of the miRNA levels shown in Figure 1A, indicated higher levels of *miR-675-5p* in SW620 metastatic cells compared to non-metastatic SW480.

In order to investigate the role of *miR-675-5p* in colon cancer progression, we silenced *miR-675-5p* with a specific inhibitor in both cell lines. QRT-PCR indicated that both SW480 and SW620 respond to miRNA inhibitor by reducing the expression of Snail and Slug (master genes of EMT); while, we found an increase of the transcriptional level of the epithelial marker E-cadherin, known as a suppressor of invasion during carcinoma progression [29] (Figure 1B). Immunofluorescence analyses, in particular in SW620 mesenchymal-like cells, showed enforced expression of E-CADHERIN and acquisition of ZO-1 in the cell membrane after treatment with miR-675-5p inhibitor; meanwhile, a reduction could be observed in nuclear SNAIL (Figure 1C). Moreover, the motility assay in Figure 1D confirmed transcriptional and proteic data showing a significative reduction in motility in SW620 cells transfected with inhibitor, compared to scrambled control. No differences were found in SW480 probably due to the reduced motility of these cells as already demonstrated by Luo et al. [30].

Overall, these data suggest a direct role of *miR675-5p* in sustaining mesenchymal phenotype and enhancing cell migration.

### *MiR-675-5p* increases EMT genes transcription by promoting HIF1α pathway

**Figure 2:**
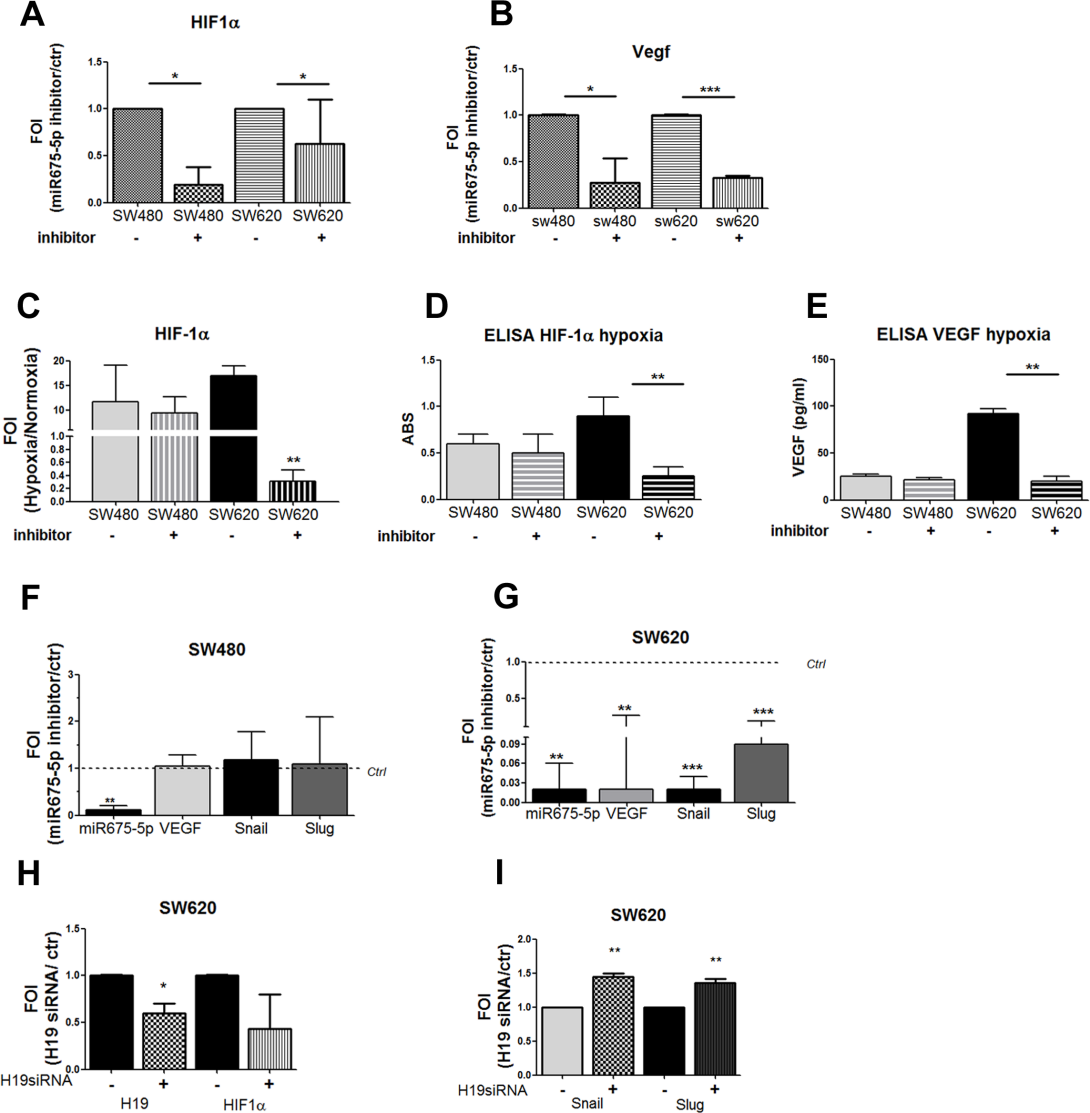
miR-675-5p activates EMT genes by promoting HIF1α pathway Real time-PCR for HIF1α (**A**) and VEGF-A (**B**) in SW480 and SW620 cells treated with miR-675-5p inhibitor or scramble control. Data were normalized for β-actin and ΔΔct is expressed as FOI with respect to expression in control samples. SW480 miRNA inhibitor *vs* SW480 control *p < 0.05; SW620 miRNA inhibitor *vs* SW620 control *p < 0.05 (**C**) Real time-PCR for HIF1α in SW480 and SW620 cells treated with miR-675-5p inhibitor or scramble control after 6 hours of hypoxia. Data were normalized for β-actin and ΔΔct is expressed as FOI with respect to expression in normoxia. Values are presented as the mean ± SD. SW620 inhibitor *vs* SW620 ctr **p < 0.001. ELISA assay for HIF-1α (**D**) and VEGFa (**E**) performed in SW480 and SW620 cells, transfected with miRNA inhibitor or scramble control, after 6 hours of hypoxia. Data are expressed as Absorbance (ABS) values at 450 nm. SW620 miRNA inhibitor *vs* SW620 scramble control **p < 0.001. Real time-PCR for hypoxia targets genes and EMT regulator genes (VEGF, SNAIL, SLUG) in SW480 cells (**F**) and SW620 cells (**G**) treated with miR-675-5p inhibitor or scramble control, after 6 hours of hypoxia. Data were normalized for β-actin and ΔΔct is expressed as FOI with respect to expression in control samples. SW620 inhibitor *vs* SW620 control **p < 0.001. (**H**) Real time-PCR for lncH19 and HIF1α in SW620 cells treated with siRNA H19 or scramble control and exposed to hypoxia for 6 hours. Data were normalized for β-actin and ΔΔct is expressed as FOI with respect to expression in control samples. SW620 siH19 *vs* SW620 control *p < 0.05;. (**I**) Real time-PCR for Snail and Slug in SW620 cells transfected with siRNA H19 or scrambled control and exposed to hypoxia for 6 hours. Data were normalized for β-actin and ΔΔct is expressed as FOI with respect to expression in control samples. **p < 0.001. Data are the mean ± SD of three independent experiments.

Recently, we demonstrated *in vitro* and *in vivo*, in a model of glioblastoma, that *miR675-5p* works as a hypoxia mimetic factor while its inhibition reduces tumour growth by affecting HIF1α stabilization [25]. Hypoxia induced EMT is a well described process in several solid tumours, and EMT genes are included in the list of HIF1α’targets [31, 32]. We investigated the possible role of HIF1α as mediator of *miR-675-5p* effects in colon carcinoma cells. As shown in Figure 2, miRNA inhibitor down regulated the expression of both HIF1α and its target VEGF in SW-cell lines (Figure 2A, 2B). In order to *i)* validate the effects of *miR-675-5p* on HIF1α and *ii)* confirm the crosstalk between *miR-675-5p*, HIF1α and EMT genes in colon carcinoma cells, we silenced *miR-675-5p* in hypoxic condition when HIF1α pathway is well established. First, we demonstrated that both cell lines respond to low O_2_ partial pressure by activating HIF1α pathway. As shown in Supplementary Figure 1, low oxygen condition induced, in both cell lines, an increase of HIF1α mRNA (Supplementary Figure 1A) and nuclear accumulation of HIF1α protein (Supplementary Figure 1B). The translocation of the hypoxic transcription factor induced VEGF gene expression and protein levels (Supplementary Figure 1C, 1D) together with HIF1α targets involved in EMT and migration: Snail and Slug (Supplementary Figure 1E). Moreover, as already found in glioblastoma, hypoxic condition induced a specific up regulation of *miR-675-5p* (Supplementary Figure 1F). This data indicated that both cell lines physiologically responded to low oxygen, with higher evidences in SW620 cells, as confirmed by transcriptional and protein analysis of HIF1α targets genes. While SW480 and SW620 cells similarly responded to hypoxic stimuli, the treatment with the miR-675-5p inhibitor in hypoxia induced different effects between cell lines. The miR-675-5p inhibitor, while it did not affect HIF1α pathway in SW480 cells (Figure 2C–2F), it completely turned off the hypoxic response in SW620, in which we found a down regulation of HIF1α, both at mRNA and protein level (Figure 2C, 2D), together with the inhibition of its targets, including EMT genes (Figure 2E, 2G). In order to attribute these effects exclusively to *miR-675-5p* we silenced the lncRNA H19 in hypoxic SW620 cells. As shown in (Figure 2H, 2I) H19 silencing did not affect HIF1α, Snail and Slug expression in SW620 cells. These data confirmed a cross talk between HIF1α, miR-675-5p and EMT, in the metastatic SW620 cells, and identified, for the first time to our knowledge, a role of *miR-675-5p* independent of the expression of its “precursor” lncH19.

**Figure 3:**
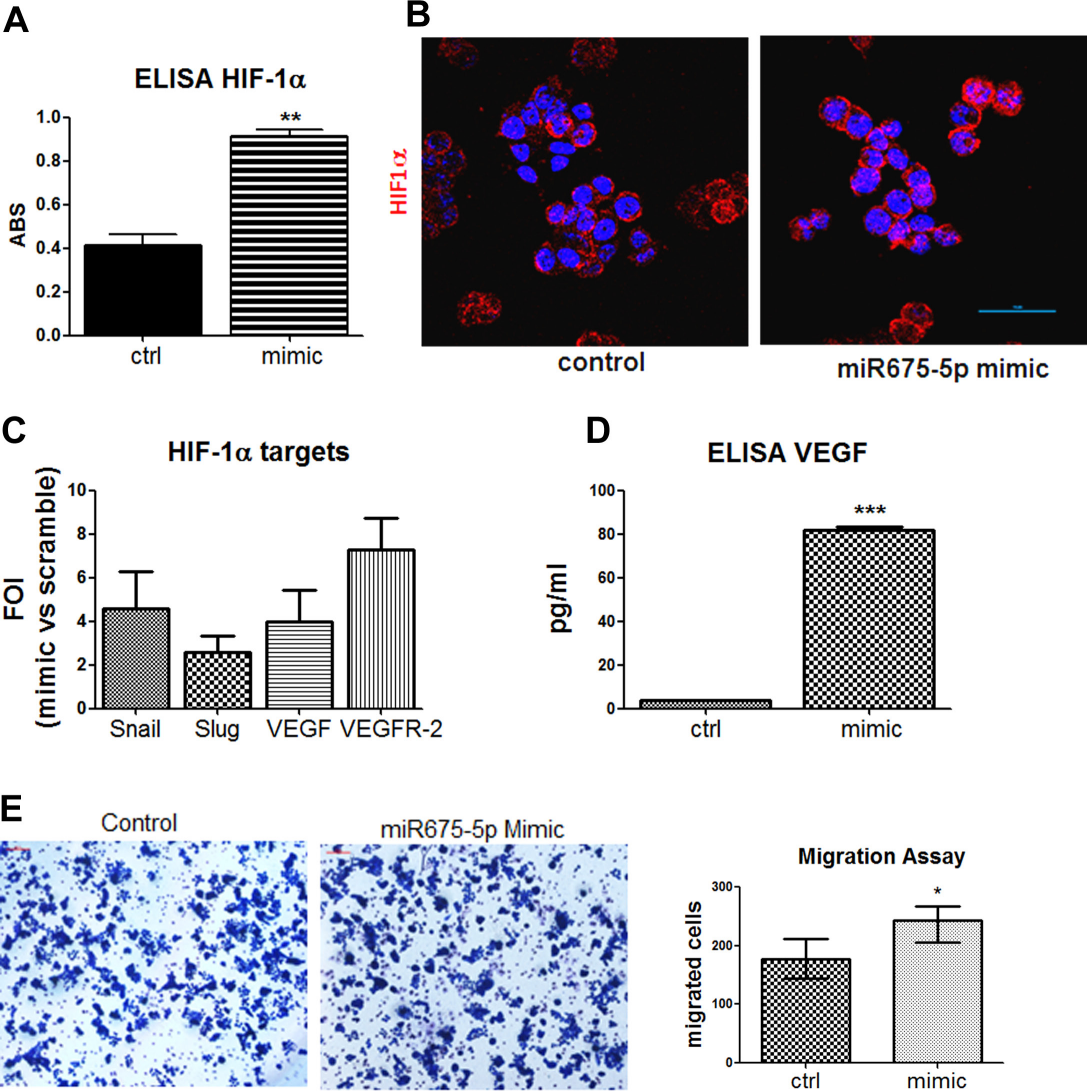
Gain of function suggests a critical role of miR-675-5p on colon cancer aggressiveness (**A**) ELISA assay for HIF-1α performed in SW620 cells nuclear extracts after mimic or scramble-control transfection. Data are expressed as Absorbance (ABS) values at 450nm. SW620 mimic *vs* SW620 scramble control **p < 0.001. Data are the mean ± SD of three independent experiments. (**B**) Immunofluorescences and median focal plane of confocal analysis for HIF1α (red) in SW620 cells treated with miR-675-5p mimic or scrambled control, in blue the nuclear staining with DAPI. (**C**) Real-time PCR for HIF1α targets gene (Snail, Slug, VEGF, VEGFR-2) from SW620 cells after miR-675-5p mimic or scramble control transfection. Data were normalized for β-actin and ΔΔct is expressed as FOI of indicated genes after mimic transfection with respect to scramble control. (**D**) ELISA assay for VEGF levels in supernatants from SW620 cell lines 18 hours after mimic and scramble control transfection. Data are expressed as pg/ml of soluble VEGF. SW620 mimic *vs* SW620 scramble control ***p < 0,0001. Data are the mean ± SD of three independent experiments. (**E**) Migration assay: Phase contrast micrographs (10×) showing the migration of SW620 cells pre-treated with mimic miR-675-5p and scramble control. Right panel: Quantification of motility established by counting the number of migrated cells (violet) per field; SW620 mimic *vs* SW620 scramble control *p < 0.05.

In the light of the obtained data, further efforts into understanding the role of *miR-675-5p* in colon cancer progression have focused on metastatic SW620 cells, in which the miRNA has demonstrated to play a dominant role. Experiments of miRNA over expression indicated that *miR-675-5p* in SW620 cells, promoted the activation of HIF1α pathway, this observation was consistent with our data obtained in glioblastoma [25]. The ELISA assay on nuclear extracts in Figure 3A and the immunofluorescence in Figure 3B, showed a nuclear accumulation of the transcription factor. As expected, nuclear increase of HIF1α allowed the transcription of its targets, as shown, in (Figure 3C, 3D). Moreover, an increase in motility has been found in SW620 cells after mimic transfection (Figure 3E).

Overall, these data indicated that *miR-675-5p*, in colon carcinoma, is able to favour hypoxia induced EMT by sustaining HIF1α pathway.

### MiR-675-5p allowed EMT gene expression by down regulating the repressor DDB2

**Figure 4:**
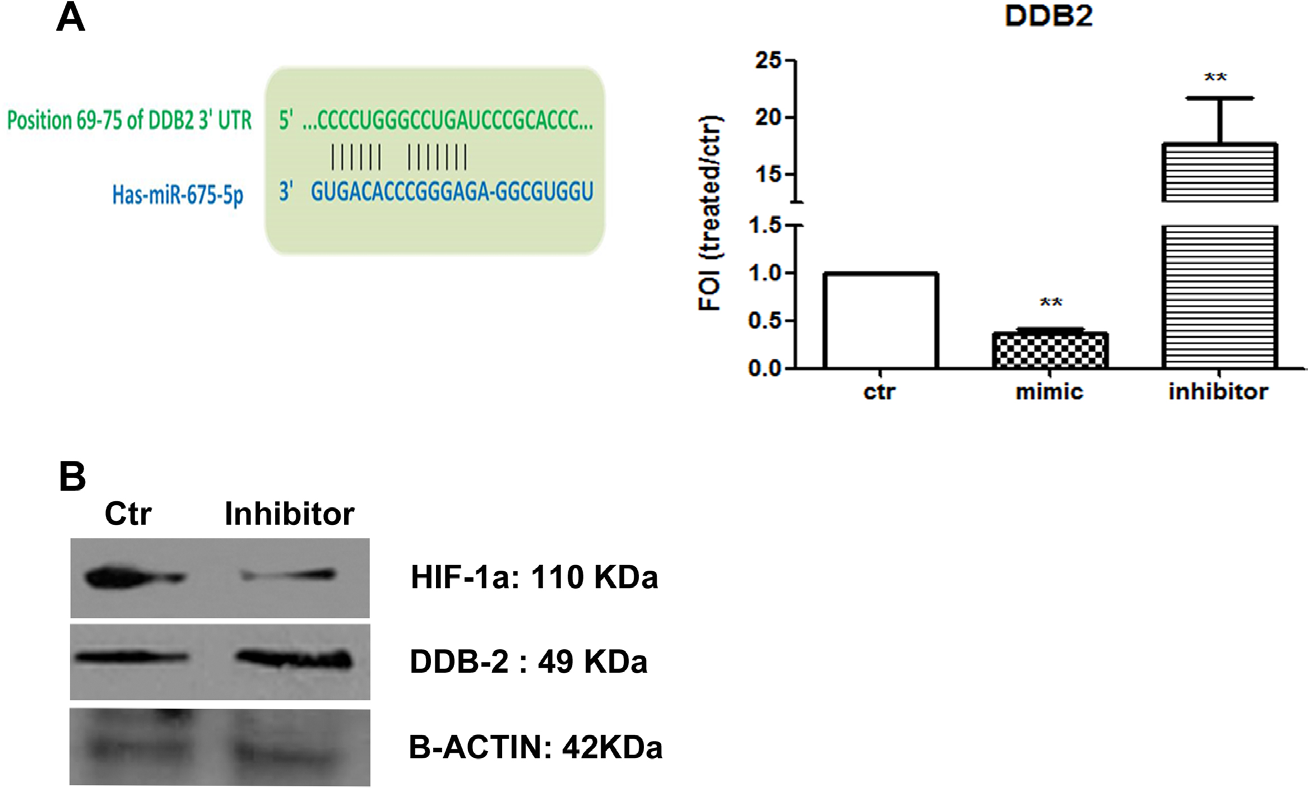
miR-675-5p down regulates the repressor DDB2 (**A**) Left: predicted seed sequence for miR-675-5p on DDB2 gene. Right: Real-time PCR for DDB-2 gene from SW620 cells after 18 hours of miR-675-5p mimic or miR-675-5p inhibitor or scramble control transfection. Data were normalized for β-actin and ΔΔct is expressed as FOI with respect to scramble control. SW620 mimic and inhibitor *vs* SW620 **p < 0,001. Data are the mean ± SD of three independent experiments. (**B**) Western blot for DDB2, HIF1α and β-actin in SW620 cells after 18 hours of miR-675-5p scramble control or inhibitor transfection.

Thanks to the analysis of miRNA targets through Target Scan, we found the damage specific DNA binding protein 2 (DDB2) listed between the putative miRNA targets (sequence of annealing is showed in Figure 4A). DDB2 has been described as down-regulated in high-grade colon cancers moreover, it plays a dominant role as repressor of EMT genes (VEGF, Zeb1 and Snail) in colon cancer cells. This data let us to suppose that *miR-675-5p* might promote metastatic phenotype by coordinating different factors involved in regulation of EMT genes. To validate DDB2 as a target for *miR-675-5p* in colon cancer metastatic cells, mRNA levels have been analysed in SW620 cells transfected with the miR-675-5p mimic or inhibitor. As shown in Figure 4A, DDB2 mRNA is inhibited by miRNA mimic while, it is over expressed in SW620 cells transfected with the miRNA inhibitor. Western blot analysis showed the dual effect caused by the miRNA inhibitor treatment in SW620 which induced the increase of DDB2 protein while reduced HIF1α (Figure 4B). These data were confirmed by western blot on nuclear extracts, which revealed also a reduction of nuclear SNAIL (data not shown). Overall, our data indicated that *miR-675-5p* promotes tumour progression in colon carcinoma regulating the expression of EMT genes by at least two independent mechanisms: the activation of the transcription factor HIF1α and the inhibition of the repressor factor DDB2.

### Metastatic colon cancer express higher level of *miR-675-5p* compared to non-metastatic

**Figure 5:**
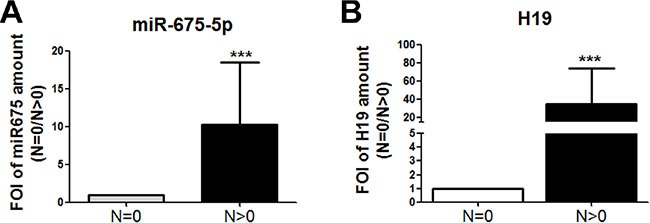
miR -675-5p expression in human colon carcinoma progression (**A**) Real time-PCR for miR-675-5p in specimens obtained from colon carcinoma patients with or without metastasis. All data were normalized for U6 and ΔΔct was expressed as FOI of analysed genes in *N* > 0 *vs N* = 0. Values are presented as the mean ± SD. ***p < 0.001 gene expression in tumour *N* > 0 *vs* tumour *N* = 0. For statistical analysis *t-test* or one- or two-way analysis of variance (ANOVA), followed by Dunnett's or Bonferroni's multiple comparison test, were performed using Prism 4(GraphPad SoftwareInc., CA, USA). (**B**) Real-time PCR for lncH19 in specimens obtained from colon carcinoma patients with or without metastasis. Data were normalized for β-actin and ΔΔct was expressed as FOI of analysed genes in *N* > 0 *vs N* = 0.

In order to validate the *in vivo* the data obtained in the *in vitro* model, the *miR-675-5p* levels were analysed in colon specimens obtained from patients with different stages of colon carcinoma. The Real time PCR in Figure 5A revealed, for the first time to our knowledge, a correspondence between lymphonode metastasis (TNM stage) and *miR-675-5p* expression. In particular, we found higher levels of *miR-675-5p* in specimens from colon cancer patients (n11) with lymphonodal metastasis (*N* > 0) compared to patients (n11) without lymphonodal compromission *N* = 0. As expected, and consistent with the data in literature, patients with lymphonodal metastasis showed also higher levels of lncRNA H19 (Figure 5B), HIF1α and its target VEGF while lower levels of DDB2 expression were found (Supplementary Figure 2).

## DISCUSSION

Colon cancer-related mortality is associated with high-grade disease, strictly connected to the number of metastasis [33]. Our data revealed a role of *miR-675-5p* in maintaining metastatic phenotype. We found, both *in vitro* and in specimens from human colon cancer patients, that metastatic colon cancer cells, overexpress *miR-675-5p* if compared to non-metastatic cells, consisting with data obtained in other tumours (glioblastoma, esophageal, squamous cell carcinoma, osteosarcoma [24, 34, 35]). Furthermore, our studies indicated that *miR675-5p* expression sustains metastatic phenotype through a hypoxia induced EMT. Matouk et al. highlighted a critical role of lncRNA H19 in breast cancer progression indicating “the axis H19/miR675 as a part of EMT programs”. They suggested that H19 may suppress E-cadherin expression by up-regulating Slug, via a mechanism that involves miR675 and which mediator has to be defined [36]. Recently, Roy et al. identified in DDB2 a suppressor of EMT for colon cancer cells with a significant decrease in high-grade colon cancer. They found that DDB2 constitutively represses EMT master genes, in SW480 cells, while its depletion, in SW620 cells, correlates with the mesenchymal phenotype [37]. Our data indicated that *miR-675-5p* promotes EMT genes expression, Snail in particular, through a dual strategy: the stabilization of a transcription factor (HIF1α) and the inhibition of a transcriptional repressor (DDB2). However, further analysis are required to better define the molecular crosstalk between these actors in order to promote the mesenchymal phenotype.

Even if colon carcinoma cell lines present a basal activation of HIF1α pathways [38], the experiments in hypoxic condition allowed to identify substantial differences between metastatic and non-metastatic cells, in response to low oxygen partial pressure. In SW620 cells, HIF1α nuclear accumulation reaches higher level compared to SW480 and this is reflected in a substantial increase of its targets genes, suggesting a greater ability of metastatic cells to activate hypoxic responses that, as known, are implicated into the promotion of angiogenesis, chemoresistance and tumour metastasis [39–41]. Our data indicated that miR-675-5p levels were increased in hypoxic condition and, only in metastatic cells, *miR675-5p* is required to maintain HIF1α pathways, thus demonstrating the existence of different control mechanisms of hypoxia between metastatic and non metastatic cells. While the inhibition of *miR-675-5p* was able, *per se*, to completely turn off hypoxic responses in SW620, it did not affect cells from primary tumour; these data enforced the concept that different strategies have to be adopted in order to treat high grade or low grade colon cancer.

## MATERIALS AND METHODS

### Cell culture and reagents

SW480 and SW620 cells were routinely maintained in RPMI supplemented with 10% heat-inactivated fetal bovine serum, penicillin and streptomycin (50 IU/ml), 2 mM glutamine (Euroclone, UK). Cells were maintained in a humidified atmosphere of 5% of CO_2_ at 37°C. To perform hypoxia experiments, both cell lines were seeded at 10,000 cells/cm^2^ and incubated in a “Hypoxic Chamber” containing 1% O_2_ gas mixture for 6 hours.

### Cell transfection

For cell transfection, Attractene Transfection Reagent (cat. number 1051531, Qiagen) was used following manufactory's indication. Briefly, SW480 and SW620 cells were seeded at 10,000 cells/cm^2^ and transfected with 15pmoles/ml of hsa-miR-675-5p inhibitor (cat. number 4464084, Life Technologies, Italy), hsa-miR-675-5p mimic (cat.number 4464066, Life Technologies) or scrambled negative control (cat. number 4464058, Life Technologies). Eighteen hours after transfection, the medium was collected and the cells processed for following assays.

### TransAM Kit

An ELISA-based kit (TransAM Kit, Vinci-Biochem, Italy) was used to detect and quantify HIF-1α transcriptional factor activity following manufacturer's instructions. Briefly, nuclear extracts were firstly prepared using the Nuclear Extract Kit (Vinci-Biochem) and 8 μg of the samples were added to the coated plate and analysed at 450 nm with Gen5 Microplate Collection & Analysis Software Data (BioTek Instruments, Inc.^®^). Data were expressed as HIF-1α protein content in total nuclear extract (Absorbance).

### ELISA assay

VEGF concentration was quantified using the ELISA kit (VEGF Human ELISA KitNovex^®^ cat. numberKHG011, Life Technologies), according to manufacturer's protocol. SW480 and SW620 -conditioned medium was collected after hypoxia treatment or transfection with miR-675-5p inhibitor or mimic. Data were expressed as VEGF concentration in pg/ml.

### RNA extraction and real-time PCR

Total RNA was extracted using the commercially available illustraRNAspin Mini Isolation Kit (GE Healthcare, Italy), according to manufacturer's instructions. Tissue RNAs was extracted using the commercially available PureLink FFPE Total RNA Isolation Kit (Invitrogen cat.number 45-7015). Human colorectal cancer specimens (*n* = 22) were collected from the pathology archives of the Human Pathology Section, Ospedali Riuniti Villa Sofia-Cervello (Palermo) in accordance with the Declaration of Helsinki and with the policy of the Institute. RNA was reverse-transcribed to cDNA using the High Capacity cDNA Reverse Transcription Kit (Applied Biosystem, USA). Real-time PCR was performed in duplicates for each data point, and the oligonucleotides used are described in Table 1.

**Table 1:** Gene primers used to study gene expression profiling

Gene	Primer forward	Primer reverse
*VEGF*	CGAGGGCCTGGAGTGTGT	CGCATAATCTGCATGGTGATG
*VEGFR*	CGGTCAACAAAGTCGGGAGA	CAGTGCACCACAAAGACACG
*H19*	GCACCTTGGACATCTGGAGT	TTCTTTCCAGCCCTAGCTCA
*HIF-1α*	TGATTGCATCTCCATCTCCTACC	GACTCAAAGCGACAGATAACACG
*E-CADHERIN*	GAGGAGAGCGGTGGTCAAAG	GTTCAGGGAGCTCAGACTAG
*SLUG*	CATGCCTGTCATACCACAAC	GGTGTCAGATGGAGGAGGG
*SNAIL*	GCGAGCTGCAGGACTCTAAT	CCCGCAATGGTCCACAAAAC
*DDB-2*	ATCAAAGGGATTGGAGCTGGAG	GCTCCATCGGGACTGAAACA
*B-ACTIN*	ATCAAGATCATTGCTCCTCCTGA	CTGCTTGCTGATCCACATCTG

Changes in the target mRNA content relative to housekeeping gene (β-actin) were determined with the ΔΔct Method. For miRNA expression, 250 ng of RNA was reverse transcripted according to manufacturer's instructions (cat.number 4366596, TaqManMicroRNA Reverse Transcription, Applied Biosystem). Taqman probes were used to analyse: miR-675-5p (cat.number 4440887, Applied Biosystem), miR-675-3p (cat.number 4427975, Applied Biosystem), U6 (cat.number 4427975 Applied Biosystem), and H19 (Hs00262142_g1 Life Technologies). Changes in the target miRNA content relative to housekeeping U6 were determined with the ΔΔct Method.

### Silencing of lncH19

SW620 cells were grown at a density of 100.000 cells/well in a 6 wells plate, and transfected by using Attractene Transfection Reagent (cat. number.1051531, Quiagen) for 6 hours with 0.2 mg/ml H19 siRNA (SR319206B Origene Technologies) or scramble negative control (SR30004 Origene Technologies) at the same dose following manufacturer's indications.

### Western blotting

SDS-PAGE and Western Blotting (WB) were performed according to standard protocols. Briefly, SW620 cells after transfection with miR-675-5p inhibitor were lysed in lysis buffer containing 15 mM Tris/ HCl pH7.5, 120 mM NaCl, 25 mM KCl, 1 mM EDTA, 0.5% Triton X100, Halt Protease Inhibitor Single-Use cocktail (100X, ThermoScientifc). Whole lysate (15 μg per lane) were separated using 4–12% NovexBis-Tris SDS-acrylamide gels (Invitrogen), electro-transferred on Nitrocellulose membranes (Bio-Rad), and immunoblotted with the appropriate antibodies. The antibodies against the following proteins were used: HIF1α (Anti-HIF 1α, Rabbit Polyclonal cat. number 1854599, Millipore), DDB2 (DDB2 (H-127): sc-25368, Santa Cruz Biotechnology, INC.), SNAIL (Snail (C15D3) Rabbit mAb#3879, Cell Signaling), and β-Actin (Monoclonal anti-β-actin, A5316 Sigma). All secondary antibodies were obtained from Thermo Fisher Scientific. Immunofluorescence was detected using ChemiDoc Biorad acquisition instrument.

### Immunofluorescence analysis

Immunocytochemistry was done on PFA 4% fixed cells, and stained with the following primary antibodies: anti-E-Cadherin (BD Biosciences 610181), anti-SNAIL (sc-28199, Santa Cruz Biotechnology, INC.) and anti-ZO-1 (ZO-1 (C-19): sc-8146, Santa Cruz Biotechnology, INC.); the secondary antibodies were Alexa-Fluor 488 and Alexa-Fluor 594, from Molecular Probes. The nuclei were stained with NucRed^®^ Live 647 (Catalog number: R37106, Life Technologies), and preparations were analysed by confocal microscopy (Nikon A1).

### Transwell cell migration assay

Cell migration assay was performed as described by [42, 43]. Briefly, 18 hours after transfection, cells were trypsinized and adjusted to 8 × 10^5^ cells/ml of cell suspension, 200 μl from this solution, were seeded into the upper chamber of transwells (Cell culture insert, Corning, cat. Number 353097), while 800 μl of medium with 20%FBS was added in the lower chamber. Cells were then cultured at 37 °C for 48 h and the cells on the surface of the up chamber were removed with cotton swaps and the cells under the surface of the low chamber were stained with crystal violet (0.1%). Migrated cells were examined under an inverted microscope and photographed at 20× magnification. The number of cells were measured manually with IMAGE-J software (http://rsbweb.nih.gov/ij/).

### Statistical analysis

*In vitro* experiments were repeated three times, giving reproducible results. Data are presented as mean values ± standard deviation (SD) of three independent experiments. For statistical analysis *t-test* or one- or two-way analysis of variance (ANOVA), followed by Dunnett's or Bonferroni's multiple comparison test, were performed using Prism 4 (GraphPad SoftwareInc., CA, USA).

## CONCLUSIONS

For the first time to our knowledge, we identified the leading role of miR-675-5p in hypoxia induced EMT, that is independent from “its precursor” the lncRNA-H19. Moreover, our data obtained from CRC specimens indicated *miR-675-5p* as putative target and new prognostic marker for CRC. However, further studies are required to identify the molecular mechanisms that increase *miR-675-5p* levels in metastatic cells.

## SUPPLEMENTARY MATERIALS FIGURES



## Abbreviations

CRC: Colorectal cancer
EMT: epithelial to mesenchymal transition
VEGF: vascular endothelial growth factor
lncRNA: long-non-coding RNA
HIF1α: Hypoxia-inducible factor 1-alpha
TNM: Classification of Malignant Tumors
ZO-1: Zonula occludens-1
DDB2: damage specific DNA binding protein 2.
