# Supplementary material for: MiR-675-5p supports hypoxia induced epithelial to mesenchymal transition in colon cancer cells
Source: Oncotarget. 2017 Jan 3;8(15):24292–302. doi: 10.18632/oncotarget.14464 (PMC5421847; doi:10.18632/oncotarget.14464)
Supplement: Supplementary file 1 [file oncotarget-08-24292-s001.pdf]

MiR-675-5p supports hypoxia induced epithelial to mesenchymal transition in colon cancer cells

Supplementary Materials

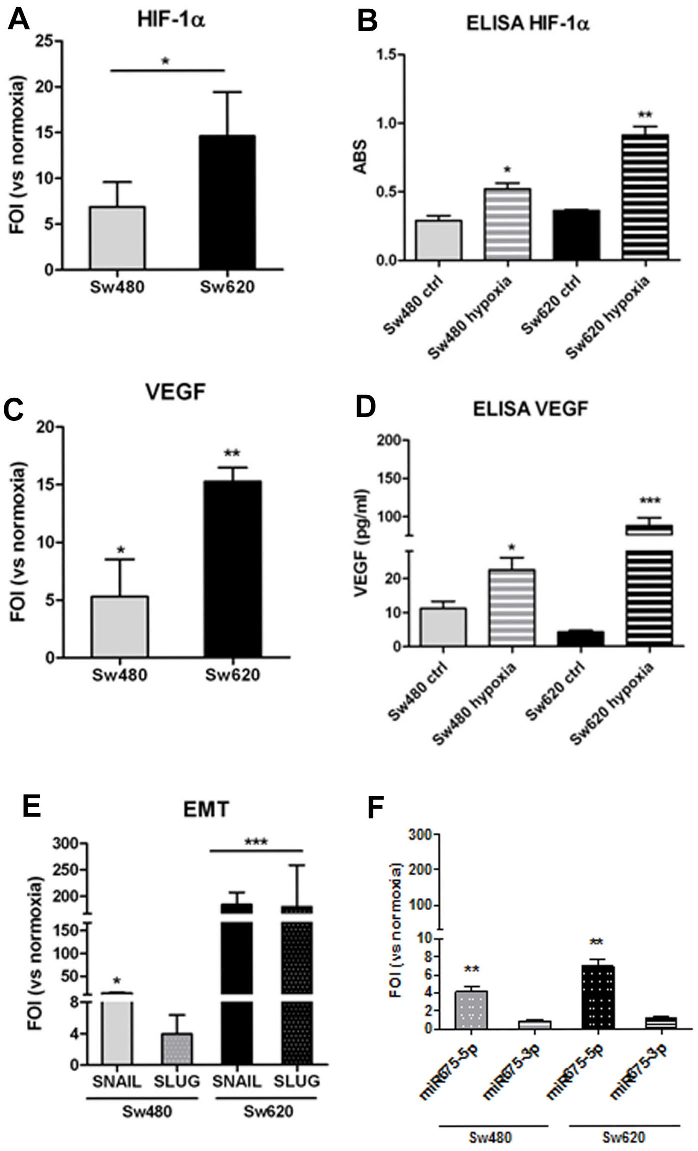

Supplementary Figure 1: SW480 and SW620 cells respond to hypoxia stimuli. (**A**) Real-time PCR for HIF1α performed on SW480 and SW620 cells. Data were normalized for β-actin and ΔΔct is expressed as FOI of analysed genes in hypoxia *vs* normoxia. SW620 *vs* SW480 *$p < 0.01$ (**B**) ELISA assay for HIF-1α performed in SW480 and SW620 cells nuclear extracts. Data are expressed as Absorbance (ABS) values at 450 nm. SW480 hypoxia *vs* SW480 *$p < 0.01$; SW620 hypoxia *vs* SW620 **$p < 0.001$ (**C**) Real-time PCR for VEGF performed on SW480 and SW620 cells. Data were normalized for β-actin and ΔΔct is expressed as FOI of analysed genes in hypoxia *vs* normoxia. SW480 hypoxia *vs* SW480 *$p < 0.01$; SW620 hypoxia *vs* SW620 **$p < 0.001$. (**D**) ELISA assay for VEGF levels in supernatants from both cell lines. Data are expressed as pg/ml of soluble VEGF. SW480 hypoxia *vs* SW480 *$p < 0.05$; SW620 hypoxia *vs* SW620 ***$p < 0.0001$ (**E**) Real-time PCR for EMT master genes: Snail and Slug, performed on SW480 and SW620 cells. Data were normalized for β-actin and ΔΔct is expressed as FOI of analysed genes in hypoxia *vs* normoxia. SW480 hypoxia *vs* SW480 *$p < 0.01$; SW620 hypoxia *vs* SW620 ***$p < 0.0001$. (**F**) Real-time PCR for miR-675-5p and miR-675-3p performed on SW480 and SW620 cells. Data were normalized for U6 and ΔΔct is expressed as FOI of analysed genes in hypoxia *vs* normoxia. SW480 hypoxia *vs* SW480 **$p < 0.001$; SW620 hypoxia *vs* SW620 **$p < 0.001$. Data are the mean ± SD of three independent experiments.

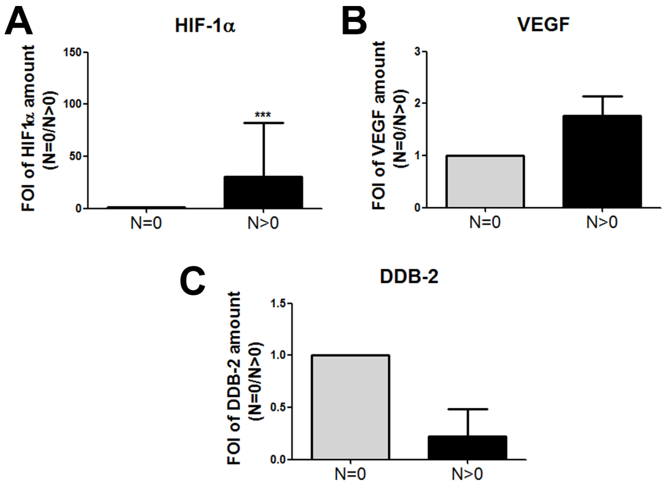

Supplementary Figure 2: miRNA 675-5p induced HIF1α pathway in human colon carcinoma metastasis. Real time-PCR for HIF1-α, VEGF and DDB-2 in specimens obtained from colon carcinoma patients with or without metastasis. All data were normalized for β-actin and ΔΔct was expressed as FOI of analysed genes in $N > 0$ *vs* $N = 0$. Values are presented as the mean ± SD. ***$p < 0.001$ for HIF-1 α gene expression in tumour $N > 0$ *vs* tumour $N = 0$.
